# Supplementary material for: The efficacy of a regimen comprising clarithromycin, clofazimine, and bedaquiline in a mouse model of chronic Mycobacterium avium lung infection
Source: Antimicrob Agents Chemother. 2025 Mar 14;69(4):e01853-24. doi: 10.1128/aac.01853-24 (PMC11963545; doi:10.1128/aac.01853-24)
Supplement: Table S1 — Statistical analysis of mean CFU burden in each treatment group. [file aac.01853-24-s0001.pdf]

SUPPLEMENTAL INFORMATION

The efficacy of a regimen comprising clarithromycin, clofazimine and bedaquiline in a mouse model of chronic *M. avium* lung infection

Binayak Rimal,[1] Ruth A. Howe, [1] Chandra Panthi, [1] Gyanu Lamichhane[1,2]

[1]Division of Infectious Diseases, Department of Medicine, School of Medicine, Johns Hopkins University, Baltimore, MD 21287, USA. [2]Center for Nontuberculous Mycobacteria and Bronchiectasis, School of Medicine, Johns Hopkins University, Baltimore, MD 21287, USA.

SUPPLEMENTARY TABLE

Table S1: Statistical assessment of lung *M. avium* burden between groups of mice receiving different treatments.

M. avium strain ID	Treatments compared	*p*-value from t-Test: Two-Sample Assuming Unequal Variances (P(T<=t) two-tail)					
		Week +1	Interpretation of *p*-value	Week +4	Interpretation of *p*-value	Week +8	Interpretation of *p*-value
M. avium 101 (Figure 1a)	PBS vs. CLR	0.07407	ns	0.000025	**	0.000000002	**
	PBS vs. CFZ	0.44730	ns	0.001783	**	0.000164074	**
	PBS vs. RFB	0.31565	ns	0.004227	**	0.000048710	**
	CLR vs. CFZ	0.16291	ns	0.930875	ns	0.132337093	ns
	CLR vs. RFB	0.10157	ns	0.000055	**	0.000000102	**
	CFZ vs. RFB	0.96099	ns	0.001487	**	0.002408809	**
M. avium 104 (Figure 1b)	PBS vs. CLR	0.03408	*	0.000003	**	0.000000302	**
	PBS vs. CFZ	0.88205	ns	0.000000	**	0.000003620	**
	PBS vs. RFB	0.96357	ns	0.178095	ns	0.735617048	ns
	CLR vs. CFZ	0.01858	*	0.140373	ns	0.926414910	ns
	CLR vs. RFB	0.08793	ns	0.000026	**	0.000000350	**
	CFZ vs. RFB	0.70165	ns	0.000004	**	0.000004194	**
M. avium 101 (Figure 2a)	PBS vs. CLR	0.000002016	**	0.000004551	**	0.000000680	**
	PBS vs. BDQ	0.000480579	**	0.000016393	**	0.000000273	**
	PBS vs. CLR+BDQ	0.000016107	**	0.000009474	**	0.000000297	**
	PBS vs. CLR+CFZ+BDQ	0.000000086	**	0.000000029	**	0.000000013	**
	CLR vs. BDQ	0.003048333	**	0.376703205	ns	0.641578549	ns
	CLR vs. CLR+BDQ	0.218955891	ns	0.860757925	ns	0.000196943	**
	CLR vs. CLR+CFZ+BDQ	0.000004130	**	0.000012390	**	0.000527740	**
	BDQ vs. CLR+BDQ	0.001760740	**	0.136738696	ns	0.002915695	**
	BDQ vs. CLR+CFZ+BDQ	0.000000362	**	0.000003747	**	0.000783718	**
	CLR+BDQ vs. CLR+CFZ+BDQ	0.000013383	**	0.000006909	**	0.142107100	ns

Results of two tailed *t*-test of lung *M. avium* MAC 101 or MAC 104 burdens in mice at the completion of 1-, 4- and 8-weeks of treatment are shown. The mean MAC 101 and MAC 104 lung burdens at 1-, 4- and 8-week timepoints following treatment are illustrated in Figures 1 and 2 of the manuscript. n=5 mice per treatment group at week +1, +4 and +8. Column 1 lists the unique identifier of the infecting *M. avium* isolates and the figures in the main manuscript where lung CFU data are plotted. Column 2 lists the pairs of treatment groups within the study that are compared. PBS: 1x phosphate-buffered saline, pH 7.4. BDQ: bedaquiline, 25 mg/kg, once daily. CLR: clarithromycin, 100 mg/kg, once daily. CFZ: clofazimine, 25 mg/kg, once daily. RFB: rifabutin, 20 mg/kg, once daily. * represents p-value ≤0.05, and ** represents p-value of ≤0.01, and were interpreted as significant. “ns” represents a p-value >0.05 that was interpreted as not significant.
